# A single-environment QTL analysis of flavor-related volatile compounds in a sweetpotato mapping population

**DOI:** 10.1007/s10681-026-03810-w

**Published:** 2026-11-03

**Authors:** Modesta Abugu, Gabriel De Siqueira Gesteira, Suzanne Johanningsmeier, Marcelo Mollinari, Bode Olukolu, Kenneth V. Pecota, Massimo Iorizzo, G. Craig Yencho

**Affiliations:** 1 Department of Horticultural Science, North Carolina State University, 214 Kilgore Hall, Box 7609, Raleigh, NC 27695 USA; 2 Bioinformatics Research Center, North Carolina State University, Raleigh, NC 27695 USA; 3 Food Science and Market Quality and Handling Research Unit, USDA-ARS, 322 Schaub Hall, Box 7624, Raleigh, NC 27695-7624 USA; 4 Department of Entomology and Plant Pathology, University of Tennessee, Knoxville, TN USA; 5 Department of Horticultural Science, Plants for Human Health Institute, North Carolina State University, Kannapolis, NC USA

**Keywords:** Sweetpotato, *Ipomoea batatas*, Flavor genetics, Volatile organic compounds, Quantitative trait loci, Genetic mapping

## Abstract

Flavor is an important trait influencing consumer preference for sweetpotato, yet little is known about the genetic mechanisms controlling the volatile organic compounds (VOCs) that may contribute to the flavor of cooked storage roots. In this study, a biparental mapping population consisting of 362 genotypes was used to perform the first genetic study of VOCs in cooked sweetpotato. VOC identification was carried out using headspace solid-phase microextraction and 2-dimensional gas chromatography coupled to time-of-flight mass spectrometry. In total, 176 VOCs were analyzed, including 27 compounds which were previously identified as predictors of different flavor attributes in sweetpotato, and 14 compounds whose identities remain unknown. A high-density phased genetic linkage map including 14,367 single nucleotide polymorphism markers was used for quantitative trait loci (QTL) mapping. A total of 85 QTLs were identified, including 2 monoterpenes and 2 apocarotenoids that have been associated with a “cooked carrot” flavor in sweetpotato. No QTLs were observed for VOCs important in the prediction of “sweetpotato” and “caramel” flavor attributes, suggesting that variation in their precursor substrates or other interacting factors may contribute to these traits. Because the population was evaluated in a single environment, multi-environment validation is required to establish QTL stability before marker development. The identified QTLs nevertheless provide a foundation for future validation and genetic improvement of sweetpotato flavor.

**Supplementary Information:**

The online version contains supplementary material available at https://doi.org/10.1007/s10681-026-03810-w.

## Introduction

Sweetpotato *(Ipomoea batatas)* is a specialty crop whose production and consumption has doubled over the past decade, driven by effective marketing campaigns and consumers’ growing recognition of its nutritional properties (George et al. 2024). They are a rich source of bioactive and antioxidant compounds, and their unique flesh colors, textures, aromas and flavors make them suitable to various culinary applications. With increasing demand, improving sweetpotato flavor has become both a breeding target and a long-term goal for the sweetpotato industry.

Flavor is a major driver of consumer preference for cooked sweetpotato (Kays 2005). It is perceived through a complex process involving the interaction of multiple sensory systems (Wang and Kays 2001). During cooking, sweetpotato storage roots generate a variety of volatile organic compounds (VOCs). When chewing, the VOCs released from the food matrix travel to the olfactory receptors in the nose, where they are perceived collectively as flavor (Chen et al. 2023).

Despite its importance to consumers, the genetic basis of sweetpotato flavor is still poorly understood. This knowledge gap is largely due to the expensive and time-consuming methods used to evaluate flavor (sensory analysis) and VOC profiles (e.g. Gas Chromatography–Mass Spectrometry- GC–MS), as well as various other factors, such as post-harvest curing, genotypes and cooking methods, that influence the composition and stability of flavor compounds (Wang and Kays 2001; Shen et al. 2024).

Cooked sweetpotato VOCs can arise from several mechanisms, including the degradation of carbohydrates and amino acids during the Maillard Reaction (MR), as well as oxidation of fatty acids, carotenoids and terpenes (Kays and Wang 2000). Over 400 VOCs were identified across 22 studies in cooked sweetpotato using GC–MS, with a few of them reported to be odor active (Abugu et al. 2025). Most recently, a sweetpotato biparental mapping population was used to identify the volatile compounds associated with 10 flavor attributes including “sweetpotato”, “caramel”, “cooked carrot”, “baked potato”, and “floral”, among others (Abugu et al. 2026). These studies established links between VOCs and flavor perception, opening opportunities to investigate the genetic mechanisms controlling VOC formation to enable DNA-based strategies to select sweetpotato varieties with desirable flavor profiles.

Recent advances in sweetpotato genomics have provided new opportunities for quantitative trait loci (QTL) analyses and DNA marker-assisted breeding (Mollinari et al. 2020; Wadl et al. 2018; Wu et al. 2018; Da Silva Pereira et al. 2020). QTL analysis has been used to study the genetic architecture of traits such as root knot nematode resistance (Fraher et al. 2024; Oloka et al. 2021), β-Carotene (BC) and starch content (Cervantes-Flores et al. 2011; Gemenet et al. 2020; Kinczyk 2019; Yada et al. 2017; Yu et al. 2014), anthocyanin content (Haque et al. 2020a, 2020b), and sugar content (Kinczyk 2019) in sweetpotato. Studies in other crops, including tomato (Martina et al. 2021), citrus (Yu et al. 2017), apples (Costa et al. 2013; Yang et al. 2023) peanuts (Zhang et al. 2023a, 2023b), and beans (Bassett et al. 2021), have identified QTLs associated with flavor traits, revealing the genetic architecture of key VOCs. However, no study has identified QTLs for VOCs in sweetpotato.

In this study, we utilized a high-resolution phased genetic linkage map alongside phenotypes for VOC profiles from a biparental population to perform the first QTL study for key flavor compounds in sweetpotato. By identifying QTLs for key flavor-associated compounds, this study lays the groundwork for developing molecular markers to screen and select for consumer-preferred flavors in sweetpotato, while providing a framework for future investigations into VOCs influenced by both genetic and environmental factors.

## Materials and methods

### Plant material

The NCDM04-001 × Covington (DC) mapping population, developed by the NCSU sweetpotato breeding program and consisting of 416 progenies (Kinczyk 2019), was used as the plant material for this study. NCDM04-001 is a high dry matter, low β-carotene (BC), low reducing sugar breeding line, whereas ‘Covington’ is a low dry matter, high BC, moderate reducing sugar cultivar (Yencho et al. 2008). The resulting DC progenies have been reported to transgressively segregate for starch, BC and sugars (Kinczyk 2019); and exhibit wide variation in volatile profiles (Abugu et al. 2026). Since its development in 2017, the population has been maintained both as greenhouse plants in disease-free conditions and as storage roots propagated from vegetative cuttings in the field. In the summer of 2023, all 416 DC genotypes were planted from vegetative 25–30 cm long tip cuttings obtained from storage roots planted (bedded) in 40.0 cm × 40.0 cm × 12.7 cm plant trays (AFLAT5, 0.48 cm bottom mesh, Anderson Pots, Portland, OR) in a plastic tunnel house. Experimental plots consisted of six plant plots with 28 cm between plants and 1.06 m between ridged rows. A purple-colored clone was planted between plots to maintain consistent growth and enable easy plot identification. The growing season for cultivars was 132 days after planting and harvest was done on October 19th, 2023. Storage roots, hereafter referred to as roots, were cured after harvest for 7 days at 29 °C and 85% relative humidity (RH) and subsequently stored in the breeding program’s specialized sweetpotato storage facility in Clinton, North Carolina, at 14 °C and 85% RH. As of February 1, 2024, the roots had been in storage for 98 days.

### Volatile compound profiling

Volatile compound profiling was performed on harvested roots using the sample preparation method reported by Abugu et al. (2026), with slight modifications. For each genotype, six roots were used to obtain core samples using a 5-mm metal cork borer (Humboldt, H-9665; Schiller Park, IL, USA). The core samples were flash-frozen in liquid nitrogen and milled into a uniform puree using a Retsch MM 400 mixer mill (Retsch Technology, Haan, Germany). Approximately 500 mg of puree was transferred into headspace vials (Wheaton Microliter, cat. no. 16–1000), mixed with 1 mL of saturated NaCl solution and 10 μL of the internal standard (0.005 ng/μL ethylbenzene-d10 solution), and sealed. Three technical replicates were prepared for a subset of samples to assess reproducibility. Samples were placed in a Gerstel MPS2 refrigerated autosampler (CTC Analytics, Switzerland; LEAP Technologies, Carrboro, NC, USA) and held at 4 °C until analysis. Samples were heated directly in sealed vials at 80 °C for 30 min prior to VOC extraction, providing standardized and uniform heat transfer while minimizing variation associated with root size, moisture, and cooking kinetics. This sealed-vial system also has the advantage of capturing the VOCs formed during cooking in a sealed space to minimize losses that occur during sample handling of baked sweetpotatoes. VOCs were subsequently extracted at 80 °C for an additional 30 min using a PDMS-Carboxen-DVB solid-phase microextraction (SPME) fiber while agitating at 500 rpm (5 s on, 2 s off). The extracted sweetpotato volatile compounds were separated using a two-dimensional gas chromatograph (GC × GC) attached to a 4D high-resolution time of flight mass spectrometer (ToFMS) (LECO Corporation, St Joseph, MI, USA). Volatile compounds were desorbed in the GC inlet (Restek, Bellefonte) at 250 °C for 15 min operated in pulsed splitless mode with a pulse pressure of 255 kPa for 1 min. The GC × GC primary oven was maintained at 40 °C for 2 min and then increased at 5 °C/min to 140 °C. The temperature ramp then increased to 10 °C/min to 250 °C and oven was held at 250 °C for 3 min. The secondary oven was run at an offset temperature of 10 °C above the temperature of the primary oven. The modulator offset was 30 °C with a 4 s 2nd-dimension separation time and a 0.80 s hot pulse time. After separation, volatile compounds were detected using an electron ionization of − 70 eV; a source temperature of 200 °C; stored mass range: 25–500 μ; and an acquisition rate: 200 spectra/s.

#### Chromatographic data processing and semi-quantitative analysis of flavor- associated VOCs

Following the exclusion of genotypes lost during harvest or storage and samples with poor-quality chromatograms, 362 samples were retained for data processing. A three-level data processing approach was used to identify the VOCs used in this study. First, a peak find method was applied to identify all peaks in the 362 genotypes using ChromaTOF software (LECO Corporation, St. Joseph, MI). Peaks were detected at a signal-to-noise ratio ≥ 10 and matched against the NIST 20 library with similarity ≥ 800 and peak width = 0.06 s. Second, an untargeted analysis and chromatogram alignment were performed using LECO ChromaTOF Tile (v.1.01, LECO Corporation, St. Joseph, MI). Sample classes included 362 genotypes (sample class) and 74 blanks, with a tile size of 10 modulations (8 s) × 45 spectra (300 ms). Peaks were identified using the same parameters as the peak find method above with RI deviation ≤ 50. Tile hits were ranked by F-ratio ≥ 20, and features more abundant in blanks were removed as artifacts. The resulting annotations (225 VOCs) were validated by manual inspection against ChromaTOF peak tables, comparing retention times, retention indices, and mass spectra. A total of 149 curated VOCs were retained for further analysis. Finally, a target analyte find method was applied for the targeted identification of flavor-associated compounds previously reported in the DC population (Abugu et al. 2026; Table S7) From that study, 27 flavor-associated compounds with identities confirmed against authentic standards were selected for inclusion, bringing the total number of identified VOCs to 176. Analyte find parameters were set using the retention times in the first and second dimensions and key mass fragments of selected compounds. All identified VOCs were collated for subsequent data analysis.

#### Data analysis

Least squares mean (LS-means) regression was used to identify the differentiating VOCs among genotypes and a Pearson correlation was subsequently performed to assess relationships among these compounds. To evaluate the distribution of phenotypic variation among progeny, frequency histograms were constructed for each differentiating VOC. These differentiating VOCs were then used for QTL mapping. All statistical and data analyses were conducted using JMP Pro 15.2.0 (SAS Institute Inc., Chicago, IL, USA) and R ‘stats’ package (R Core Team, 2022), ‘ggcorrplot’ package (version 0.1.4.1) (Kassambara 2016), ‘factoextra’ (version 1.0.7) (Kassambara and Mundt 2016) and ‘ggplot2’ package (version 3.4.4) (Wickham et al. 2007).

### QTL analysis

The construction of the DC genetic linkage map was described by Kinczyk (2019). In summary, high-quality GBS data was generated using a GBS protocol adapted for polyploids (GBSpoly, Rickman et al. 2025). Sequence reads were aligned against the I. *trifida* reference genome, and variants and genotypes were called using TASSEL4-Poly and SuperMASSA (Serang et al. 2012; Pereira et al. 2018). Genotype dosages were filtered for quality control attributes, and pairwise recombination fractions were calculated and used for grouping and ordering of markers within chromosomes. Final linkage phases and recombination fractions were jointly re-estimated for the 15 linkage groups (LGs) using a multi-point hidden Markov model (HMM, Mollinari and Garcia 2019), and final genotype probability distributions used as input for the QTL mapping step.

The resulting high-resolution phased genetic linkage map was based on 14,367 SNP markers distributed among 15 linkage groups (LGs), covering 2,727.1 cM of the hexaploid sweetpotato genome. The distribution of dosage combinations in the parents NCDM04-001 and ‘Covington’ was composed primarily of 51.5% simplex markers (present in a single copy in one parent and absent in the other, denoted as 0–1, 1–0), representing the most abundant form of SNPs in the sweetpotato genome. The average length of the linkage groups was 181.8 cM, with the longest being LG 1 at 243.0 cM, and the shortest being LG 8 at 120.5 cM. Final HMM-based conditional genotype probabilities were calculated and used as input for the QTL mapping step.

The QTLpoly software v. 0.2.4 (https://gabrielgesteira.github.io/QTLpoly/), which provides a random-effect multiple interval mapping framework for QTL mapping in polyploids ( Pereira et al. 2020), was used to detect significant QTL of all differentiating VOCs. The conditional genotype probabilities obtained from the genetic linkage map were calculated every 1 cM and used as the source of genetic variation. The VOC peak areas were transformed using a log_10_ function and used as the phenotypic information in the QTL mapping procedure. Briefly, the procedure searched for putative QTL at every position along the genome based on a stepwise approach, where variance components were tested using score statistics (Qu et al. 2013). First, a null-QTL score distribution was built based on a resampling method with 1000 iterations. The 2th and 5th percentiles of this distribution were used to determine the* p*-value thresholds for the forward search and backward elimination in the QTL search algorithm. The stepwise procedure followed, where a forward search was used to add one QTL at a time under a less conservative threshold (*p *< 0.0006), followed by a backward elimination procedure where each QTL was tested against a more conservative threshold (*p *< 0.0001). In the backward elimination process, QTLs that fell below the significance threshold or those whose positions changed were dropped from the model. These procedures were repeated until model stabilization, where no QTL was added nor dropped from the model. Based on the final QTL model for each VOC, QTL positions were refined, and their effects jointly estimated, along with associated variance components and heritabilities. Finally, genes within selected QTL intervals were functionally annotated using the sweetpotato reference genome (https://sweetpotato.uga.edu/), and prioritized based on their predicted roles in VOC biosynthesis, proximity to QTL peaks, and functional relevance reported in published literature.

## Results

### Phenotypic variation of volatile compounds in the DC population

This study measured cooked storage root volatile compound profiles in the DC mapping population from a single growing season. A total of 176 VOCs were identified, all of which showed sufficient variation among genotypes to differentiate them in least squares regression. The 176 differentiating VOCs were categorized according to their likely mechanisms of formation. Sesquiterpenes were the most abundant class (26%), followed by monoterpenes (19%) and compounds derived from fatty acid oxidation (14%). Compounds with uncharacterized synthetic mechanisms accounted for 13% of all VOCs, while those likely derived from the Maillard reaction, Strecker degradation and BC degradation comprised 8%, 5% and 3% respectively (Fig S1).

**Fig. 1:**
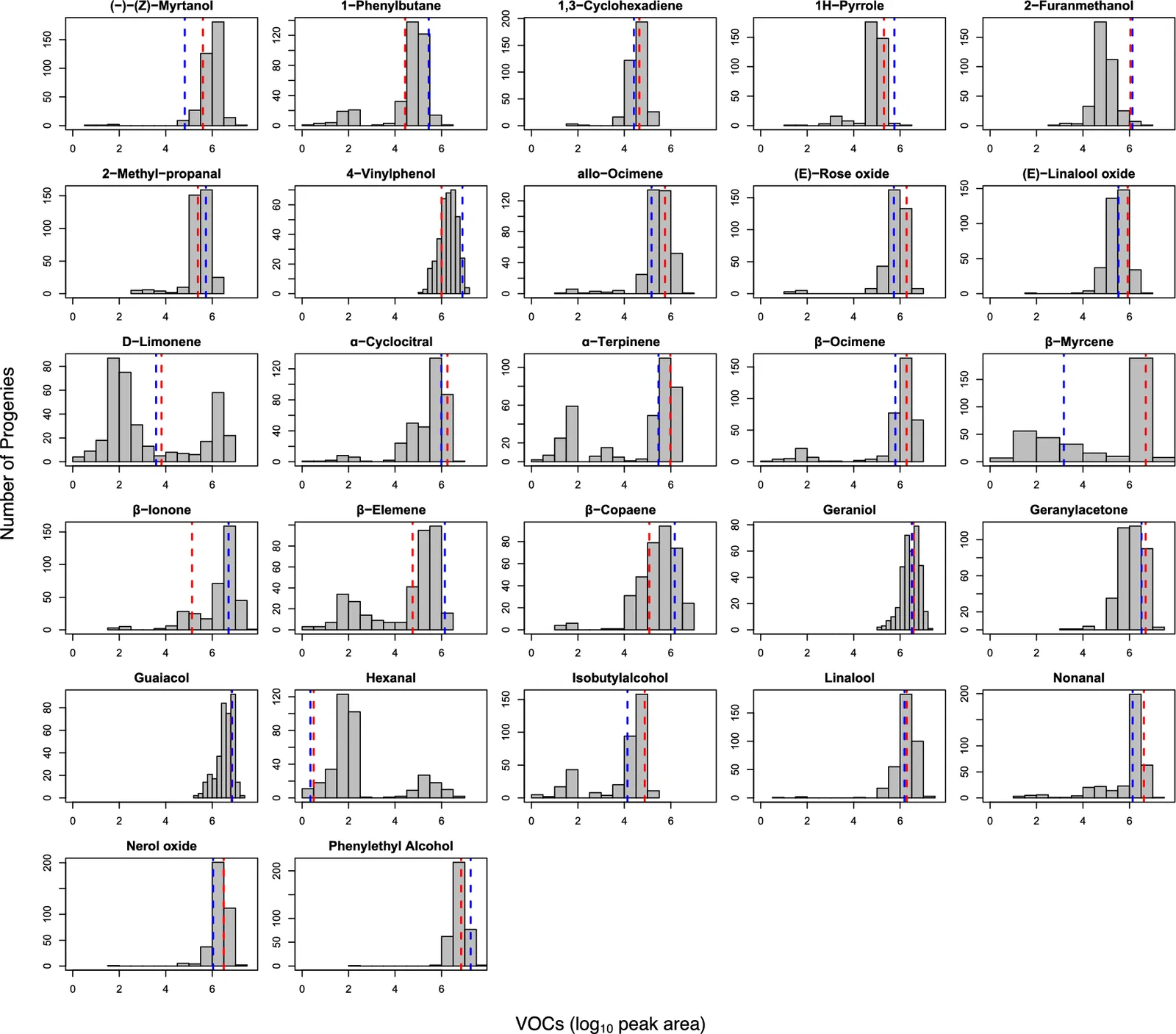
Frequency distribution of 27 flavor-associated VOCs in the DC population. Gray bars represent the frequency of progeny for a given VOC abundance level, with the x-axis indicating the abundance of VOCs (log10 peak area) and the y-axis showing the number of progenies. Red and blue dashed lines indicate the parental values for ‘Covington’ and NCDM04-001, respectively. The histograms illustrating the distributions of VOC abundance for the rest of the compounds are included in Fig S2

Frequency histograms were generated to evaluate phenotypic variation among progeny for the differentiating VOCs (Fig. S2). Of the 176 volatile compounds analyzed, 23 (~ 13%) exhibited a likely transgressive segregation pattern, with progeny showing values beyond the range of both parental lines. Distribution patterns varied across compounds, with some VOCs exhibiting relatively symmetrical distributions, whereas others displayed bimodal distributions. For example, several compounds, including 1,2-dihydrolinalool, 1-pentanol, and acetoin, exhibited relatively symmetrical distributions, consistent with the contribution of multiple genes and additive genetic effects. In contrast, a separate group of compounds, including (E)− 2-heptenal, β-cubebene, ( −)-cyperene, ( + )-(E)-calamenene, 1-(4-methoxyphenyl)− 1-pentanone, 2-butanone, and 2-butenal, displayed bimodal distributions, suggesting the possible contribution of major-effect loci or alleles with relatively large effects. Other compounds, such as methyl alcohol and 2-carene, exhibited irregular distribution patterns, potentially reflecting differences in genetic architecture, environmental effects, or other sources of phenotypic variation. These diverse distribution patterns highlight the complexity of VOC accumulation in the population. Furthermore, frequency histograms were evaluated for the 27 flavor-associated compounds (Fig. 1). With the exception of D-limonene and hexanal, most flavor-associated compounds exhibited left-skewed distributions, indicating that most progeny accumulated moderate to high levels of these compounds, whereas relatively few progeny had substantially lower concentrations (Fig. 1). Overall, the variation in segregation patterns and distribution shapes across compounds highlights the heterogeneous genetic architecture underlying VOC accumulation and supports the suitability of these traits for QTL mapping and downstream genetic dissection.

### Correlation analysis for differentiating and flavor-associated VOCs in DC population

Correlation analysis among the 176 differentiating VOCs revealed that several groups of compounds from shared pathways exhibited similar correlation patterns. For example, 39 sesquiterpenes were significantly positively correlated (*p* < 0.01) with each other and were grouped together in the correlation heatmap (Table S3). The strongest correlations (r > 0.80) were observed between (-)-germacrene D and β-copaene, aciphyllene and 6,9-guaiadene, and rotundene and cypera-2,4-diene. A similar correlation pattern was observed among the monoterpenes, where the strongest positive correlations occurred between isomers of the same compound, including (Z)-linalool oxide and (E)-linalool oxide (r = 0.98), 1,2-dihydrolinalool and dihydrolinalool (r = 0.98), and β-ocimene and allo-ocimene (r = 0.80). Strong positive correlations were also observed among selected apocarotenoids and monoterpenes, including α-cyclocitral and carvacrol (r = 0.89), dihydro-β-ionone and carvacrol (r = 0.72), and dihydro-β-ionone and α-ionene (r = 0.83). In contrast, several negative correlations were observed, including D-limonene and α-terpinene (r = − 0.48), β-cubebene and pyridine (r = − 0.45), D-limonene and pyridine (r = − 0.40), α-terpinene and β-cubebene (r = − 0.33), α-terpinene and (−)-cyperene (r = − 0.32), D-limonene and rotundene (r = − 0.31), and α-terpinene and β-elemene (r = − 0.29). Most compounds derived from Maillard reaction (MR), Strecker degradation (SD), and fatty acid oxidation (FAO) exhibited weak or inconsistent correlations, both within and among groups (Table S3).

**Fig. 2:**
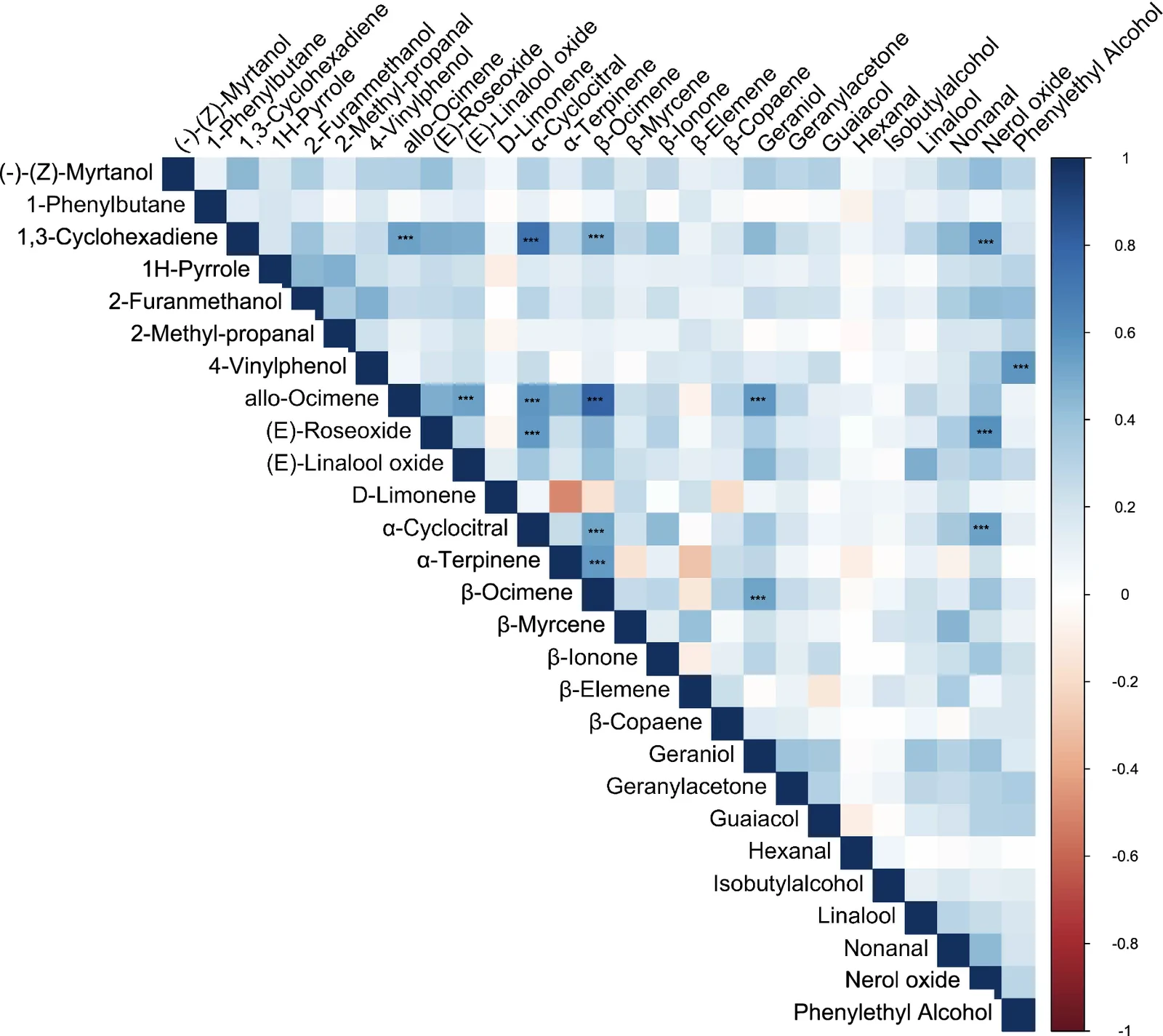
Correlation heatmap of 27 flavor-associated VOCs in the DC mapping population. Blue indicates a positive correlation and red indicates a negative correlation. The color intensity represents the strength of the correlation,*** represents correlation coefficients ≥ 0.05, and *p* < 0.05

A correlation heatmap of the 27 flavor- associated compounds in the DC sweetpotato population revealed predominantly weak or negligible relationships, with only a few notable positive associations (Fig. 2). The strongest positive correlation (r ≥ 0.81, *p* < 0.05) was observed between 1,3-cyclohexadiene and α-cyclocitral, both of which have been associated with cooked carrot flavor. Moderate positive correlations (r ≥ 0.73, *p* < 0.05) were observed among several VOC pairs, including (E)-rose oxide and nerol oxide, both associated with baked potato flavor; rose oxide and α-cyclocitral, associated with baked potato and cooked carrot flavors, respectively; and nerol oxide and α-cyclocitral, associated with baked potato and cooked carrot flavors, respectively. Additional moderate correlations were observed between β-ocimene, associated with floral flavor, and α-terpinene, associated with sweetpotato flavor; between allo-ocimene and geraniol, both associated with cooked carrot flavor; and between phenylethyl alcohol and 4-vinylphenol. Weak positive correlations (r = 0.5, *p* < 0.05) were observed between 1,3-cyclohexadiene and β-ocimene, allo-ocimene and (E)-linalool oxide, and β-ocimene and geraniol. Overall, these correlation patterns suggest that some co-occurring VOCs may reflect shared biochemical pathways, while their distinct contributions to flavor perception may arise from differences in odor thresholds and interactions among compounds within the flavor matrix.

### QTLs associated with differentiating and flavor-associated VOCs in the DC population

The 176 differentiating VOCs in the DC population were subjected to QTL analysis. A total of 85 QTLs (*p* < 0.00001) were detected for 60 VOCs in 10 linkage groups (LG2, LG3, LG5, LG6, LG9, LG10, LG11, LG12, LG13, LG14) (Table 1 and Fig. S3). Among the QTLs, 30 were detected for sesquiterpenes, 16 were for monoterpenes, 5 for carotenoid-derived VOCs (apocarotenoids), while only 1 QTL was detected for VOCs derived from SD of amino acids and 3 for VOCs derived from fatty acid oxidation. Twenty-one QTLs were associated with compounds whose precursors are currently unknown (uncharacterized), and 10 QTLs were associated with compounds that have not been previously reported in mass spectral databases (unknowns) (Table S3). Each QTL explained between 8–39% of the total phenotypic variance. On LG 3, QTLs for two apocarotenoids (α-ionene and α-cyclocitral), two monoterpenes (β-terpineol and ( + )- Z, E-nepetalactone) and three uncharacterized compounds (2-amino-4-acetamino anisole, 3-methoxy-2,5,6-trimethyl- phenol and 3,4-dimethylbenzyl alcohol) exhibited the highest percentage phenotypic variation (PVE ≥ 30%).

**Table 1:** Summary statistics of QTLs for VOCs in the DC mapping population. LOP represent the logarithm of p-value score for every genomic position significantly associated with each trait. PVE represent the percentage phenotypic variation explained by each QTL

Volatile Organic Compound	QTL No	LG No	Position (cM)	Support Interval (cM)	Marker ID	LOP	PVE (%)
*Sesquiterpenes*							
isocaryophyllene	1	2	155.64	109.42–161.31	S2_4439568	5.38	12
	2	5	127.09	83.13–132.07	S5_22057183	5.54	18
	3	6	32	16.42–41.38	S6_1411677	3.94	9
α-selinene	1	2	155.64	95.17–160.13	S2_4439568	4.16	11
	2	5	106.11	77.45–132.07	S5_20380562	5.07	18
β-selinene	1	2	155.64	101.1–182.01	S2_4439568	4.45	11
	2	5	126.17	71.16–143	S5_21957309	4.52	16
humulene	2	5	46.02	42.6–108.12	S5_4598092	5.49	16
α-gurjunene	1	5	56.03	40.34–93.01	S5_6288852	5.31	16
β-cadinene	1	5	85.1	47.02–132.07	S5_18850060	4.12	12
	2	6	30.89	22.74–34.01	S6_4245030	5.83	11
	3	14	118.06	115.08–124.35	S14_16553624	5.63	11
β-panasinsene	1	5	126.17	78.48–143	S5_21957309	4.04	18
γ-dehydro-ar-himachalene	1	5	87.13	44.3–132.07	S5_19044826	4.55	14
dehydro-ar-ionene	1	5	87.13	45.26–132.07	S5_19044826	4.76	14
γ-elemene	1	5	72.41	42.6–87.13	S5_16273303	6.21	18
aciphyllene	1	5	56.03	41.24–89.26	S5_6288852	5.82	17
guaiazulene	1	5	87.13	42.6–108.12	S5_19044826	5.71	15
cadina-1(10),6,8-triene	1	5	56.03	42.6–130.13	S5_6288852	4.75	13
(Z)-calamenene	1	5	87.13	48.43–129.13	S5_19044826	4.93	17
rotundene	1	5	105.43	78.48–147.6	S5_20286795	4.25	16
6,9-guaiadene	1	5	70.33	40.34–87.13	S5_15007205	5.70	17
aristolediene	1	5	101.21	68.26–151.03	S5_19891114	4.39	16
β-copaene	1	6	30.89	22.74–37.03	S6_4245030	5.62	11
	2	13	96.14	87.33–101.23	S13_18305474	5.88	16
(-)-germacrene D	1	6	30.89	28.16–33.01	S6_4245030	15.65	27
cyclosativene	1	6	32	31.9–34.01	S6_1411677	15.65	31
espatulenol	1	6	33.01	27.65–41.38	S6_12584417	3.97	11
α-cubebene	1	13	97.03	90.22–102.68	S13_18501410	6.01	17
β-cubebene	1	13	97.03	90.22–102.68	S13_18501410	6.01	17
*Monoterpenes*							
β-terpineol	1	3	26.25	8.11–34.07	S3_2516973	9.15	33
	2	12	168	167.02–170.12	S12_22117539	5.84	13
( + )-Z,E,-nepetalactone	1	3	9.05	8.11–31.12	S3_829608	9.23	31
	2	12	168	168–180.09	S12_22117539	5.38	12
camphene	1	3	16.26	8.11–40.44	S3_1308600	7.46	25
	2	12	168	167.02–181.09	S12_22117539	7.81	16
p-thymol	1	3	26.25	7.11–35.02	S3_2516973	8.02	29
	2	12	168	168–170.12	S12_22117539	6.66	16
geraniol	1	9	58.01	33.29–68.1	S9_3113917	5.08	17
2-carene	1	3	8.11	3.09–30.02	S3_764435	7.67	29
carvacrol	2	3	34.07	3.09–42.31	S3_3318347	6.21	24
linalool	1	9	58.01	0.59–58.01	S9_3113917	6.69	21
(E)-linalool oxide	1	9	58.01	30.24–78.01	S9_3113917	6.82	21
(Z)-linalool oxide	1	9	58.01	30.24–78.01	S9_3113917	6.67	21
allo-ocimene	1	9	44.1	0.59–78.01	S9_2217660	4.38	16
(E)-rose oxide	1	10	148.51	146.06–178.55	S10_23066140	4.70	14
*Apocarotenoids*							
β-ionone	1	3	8.11	3.09–33.02	S3_764435	6.71	24
	2	12	91.12	60.06–168	S12_6681650	3.95	09
α-ionene	1	3	8.11	7.11–28.27	S3_764435	15.65	39
α-cyclocitral	1	3	12.11	8.11–29.15	S3_994093	15.65	35
	2	12	168	168–184.15	S12_22117539	6.80	14
*Strecker degradation*							
phenylacetaldehyde	1	2	80.08	63.19–141.23	S2_10801073	3.98	39
*Fatty acid oxidation*							
6-methyl-2-heptanol	1	3	168	165.63–176.82	S3_2516973	9.80	13
	2	12	26.25	10.02–34.07	S12_22117539	5.86	29
3,4-dimethyl-1-pentanol	1	11	83.61	59.22–151.16	S11_5444504	4.00	15
*Unknowns*							
phenol, 3-methoxy-2,5,6-trimethyl-	1	3	27.87	8.11–34.07	S3_2575469	15.65	39
	2	12	168	80.16–170.12	S12_22117539	6.19	12
3,4-dimethylbenzyl alcohol	1	3	27.87	8.11–34.07	S3_2575469	15.65	38
	2	12	168	89.01–172.04	S12_22117539	5.35	11
3,3-dimethylcyclohexanone	1	3	12.11	6.02–34.07	S3_994093	15.65	38
	2	12	168	168–180.09	S12_22117539	8.33	15
3,6,6-trimethyl-cyclohex-2-enone	1	3	14	5.06–34.07	S3_1179682	10.06	33
	2	12	168	168–176.82	S12_22117539	7.34	16
1,3-cyclohexadiene	1	3	30.02	3.09–40.44	S3_2945631	4.57	23
2,3,4,5-tetramethyl-2-cyclopenten-1-one	1	3	12.11	7.11–34.07	S3_994093	15.65	38
	2	12	168	168–181.09	S12_22117539	6.61	12
2,3,4,5-tetramethylcyclopent-2-en-1-ol	1	3	8.11	0.32–39.22	S3_764435	15.65	39
	2	12	168	168–184.15	S12_22117539	6.95	13
1-(4-methoxyphenyl)− 1-pentanone	1	3	9.05	5.06–40.44	S3_829608	6.87	29
2-amino-4-acetamino anisole	1	3	26.25	7.11–35.02	S3_2516973	15.65	40
	2	12	168	168–170.12	S12_22117539	7.23	14
3-ethyl-4-methylpentan-1-ol	1	11	83.61	61.71–157.15	S11_5444504	9.94	15
2-methyl-4-(2,6,6-trimethyl-1-cyclohexenyl)- but-3-enal	1	3	9.05	0–33.02	S3_829608	8.71	32
10-methylundec-3-en-4-olide	1	3	6.02	0–28.27	S3_546844	7.53	28
1,1’-(1,1,10,10-tetramethyl-1,10-decanediyl) bis[3,4-dimethyl- cyclohexanone, 2,2,6-trimethyl-benzene	1	3	14	4.07–34.07	S3_1179682	7.15	31
	1	5	105.43	77.45–129.13	S5_20286795	4.85	17
peak 194	1	3	8.11	4.07–17.11	S3_764435	6.34	19
	2	12	168	67.08–203.49	S12_22117539	4.39	12
peak 479	1	3	8.11	1.27–34.07	S3_764435	6.19	23
peak 787	1	3	14	4.07–34.07	S3_1179682	8.56	32
	2	12	168	150.23–186.21	S12_22117539	4.51	11
peak 2316	1	3	26.25	4.07–35.02	S3_2516973	9.16	31
	2	12	168	91.12–170.12	S12_22117539	5.06	12
peak 504	1	5	106.11	42.6–129.13	S5_20380562	5.33	16
peak 958	1	5	72.41	42.6–107.18	S5_16273303	4.31	13

Table 1 summarizes all QTLs identified in this study, including 11 QTLs corresponding to eight of the 27 flavor-associated VOCs. β-Ionone and α-cyclocitral, both associated with cooked carrot flavor, each had two QTLs, with one located on LG 3 and the other on LG 12. Two QTLs were also identified for β-copaene, associated with pumpkin/squash flavor, with one QTL on LG 6 and the other on LG 13. One QTL each was identified on LG 9 for geraniol, linalool, (E)-linalool oxide, and allo-ocimene, all associated with cooked carrot flavor. An additional QTL was identified on LG 10 for (E)-rose oxide, which was associated with baked potato flavor. In contrast, no QTLs were detected for Maillard reaction-derived compounds, such as 2-furanmethanol, which have previously been associated with sweetpotato and caramel flavors. Together, these findings suggest that the genetic regulation of flavor-associated VOCs may be compound- and pathway-specific, with detectable QTLs for selected terpenoid and apocarotenoid compounds but not for all VOC biosynthetic pathways.

### QTL analysis revealed multiple overlapping hotspots

**Fig. 3:**
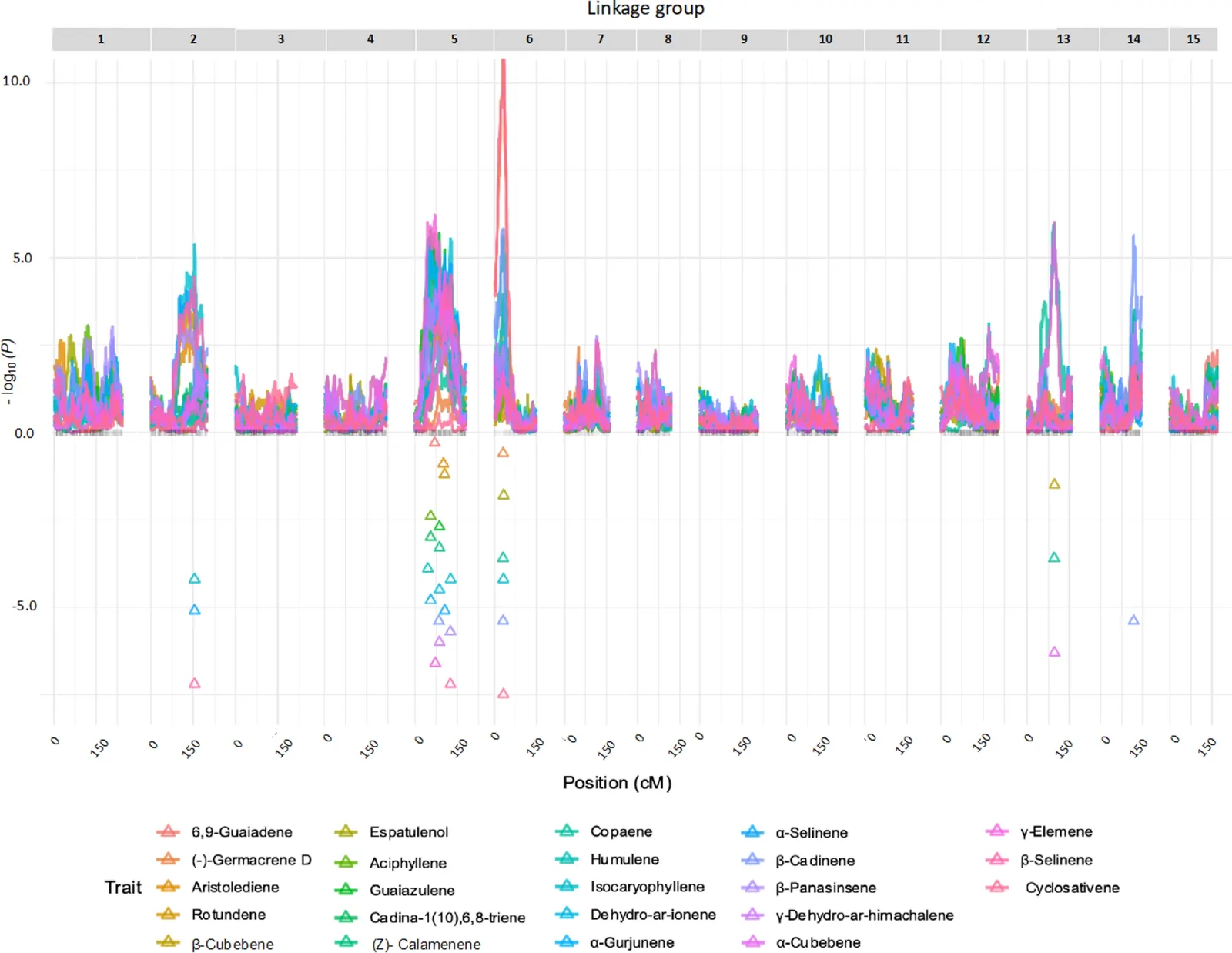
Plot showing the sesquiterpene QTLs in the DC population. -Log_10_(P) represents the logarithm of P values for every genomic position using the final QTL model for each trait

**Fig. 4:**
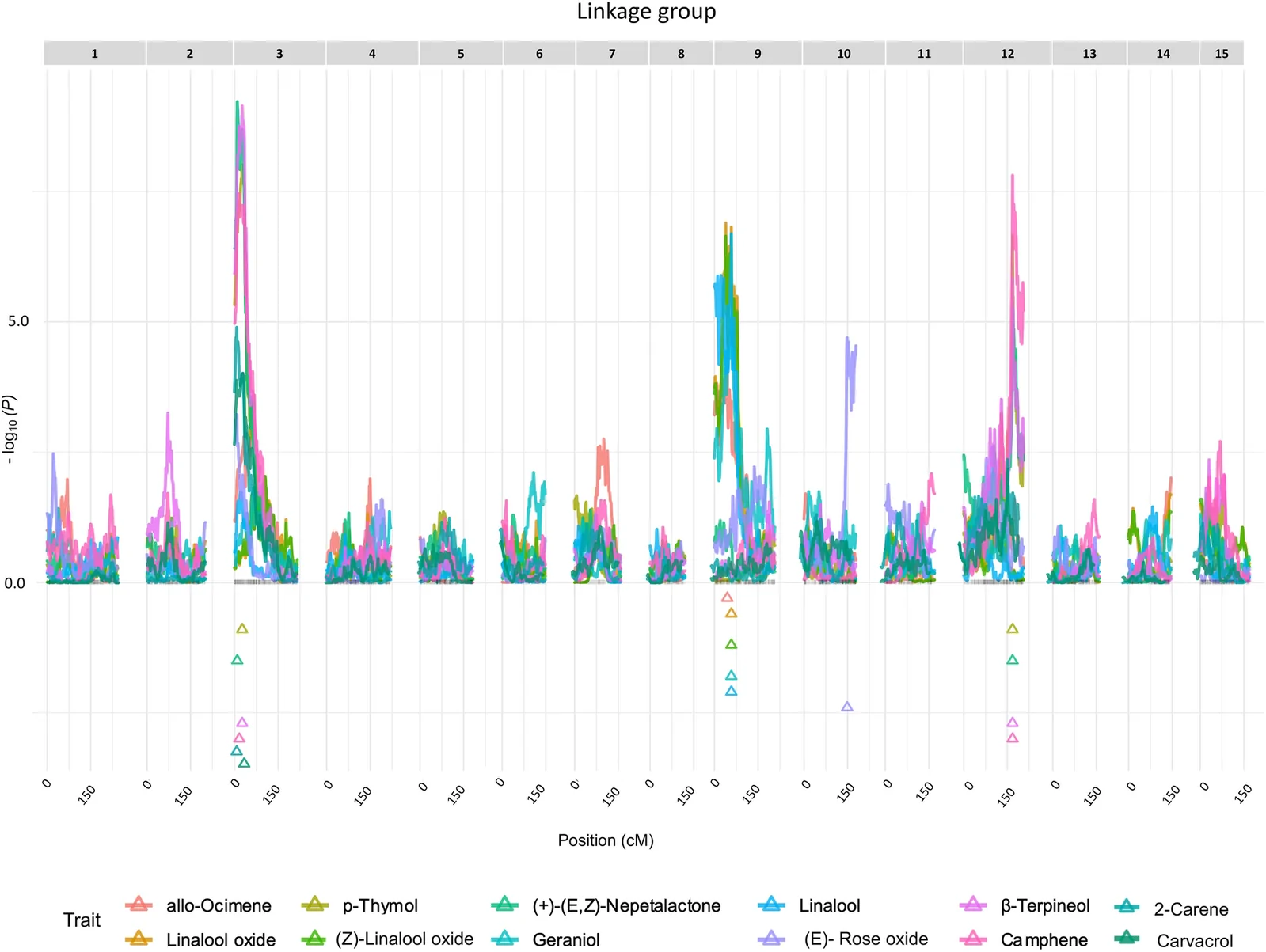
Plot showing the monoterpene QTLs in the DC population. -Log_10_(P) represents the logarithm of P values for every genomic position using the final QTL model for each trait

**Fig. 5:**
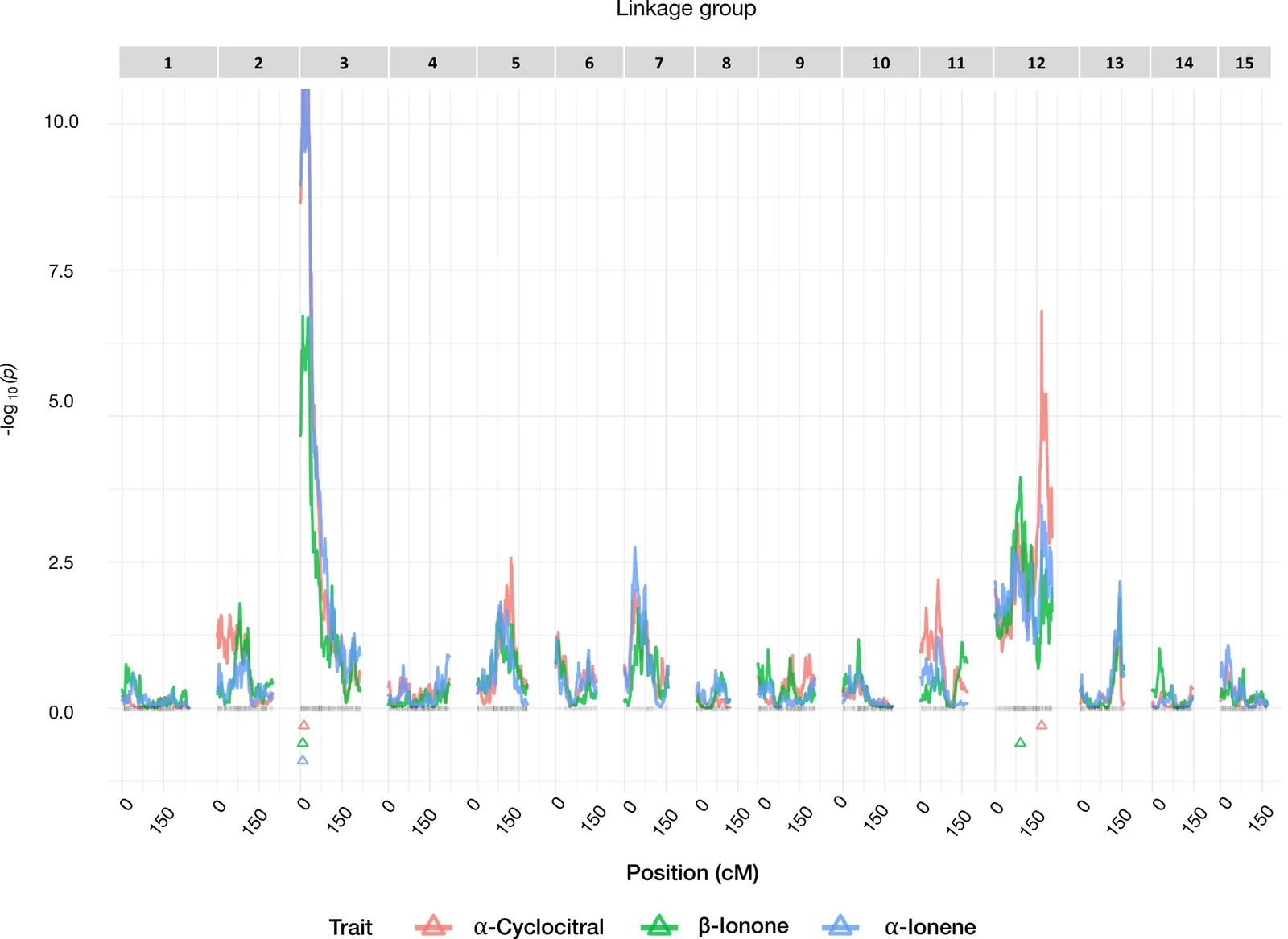
Plot showing the QTLs for apocarotenoids in the DC population. -Log_10_(P) is the logarithm of *P* values for every genomic position using the final QTL model for each trait

Several hotspots for overlapping QTLs were detected for VOCs sharing similar precursors, indicating the possibility of highly linked genes or a shared biosynthetic pathway. For example, 17 overlapping QTLs for sesquiterpenes were detected on LG 5 (Fig. 3), 7 overlapping QTLs for the monoterpenes were detected on LG 9 (Fig. 4) while 3 overlapping QTLs were detected for the apocarotenoids on LG 3 (Fig. 5). Hotspots for 12 and 7 overlapping QTLs were also detected for the uncharacterized and unknown compounds on LGs 3 and LG 12, respectively (Figs. S4, S5).

**Fig. 6:**
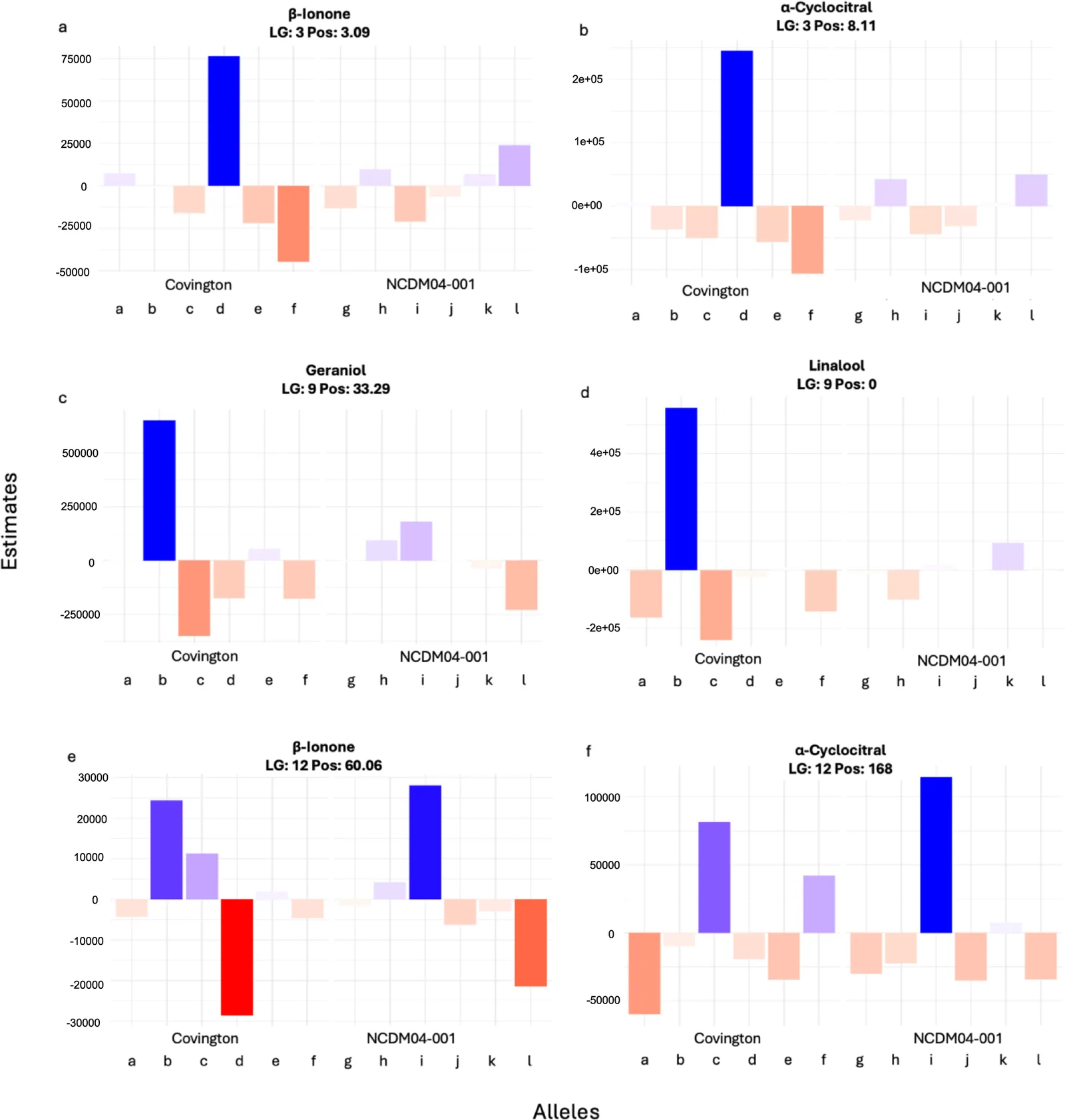
Representation of additive allelic effects for QTLs in LG 3, 9 and 12 derived from the parents, ‘Covington’ and NCDM04-001. Letters a through f, and g through l represent the six haplotypes for QTLs derived from ‘Covington’ and NCDM04-001, respectively. Positive effects (blue) indicate increased trait values, while negative effects (red) indicate decreased trait values

The overlapping QTLs spanned large support intervals in similar genomic regions for different traits, showing a possibility of genes for shared pathways. For example, the support interval for the QTLs detected for the apocarotenoids (β-ionone, and α-cyclocitral) on LG 3, ranged from 3.09–33.03 cM (markers S3_764435–S3_3261239 bp) (Table 1). Similarly, four monoterpenes (β-terpineol, ( + )-Z, E-nepetalactone, camphene, p-thymol) overlapped the same region as the apocarotenoid (Table 1), suggesting the possibility of shared genetic control or coordinated regulation of these compounds, potentially reflecting their involvement in related biosynthetic pathways. The support interval of the overlapping QTLs for five monoterpenes (representing geraniol, linalool, (E)-linalool oxide and (Z)-linalool oxide, and allo-ocimene) on LG 9 ranged between 0−78.01 cM (marker S9_59546–S9_4038847 bp) (Table 1). On LGs 3 and 9, ‘Covington’ contributed major alleles (‘d’ for LG 3 and ‘b’ for LG 9) for increasing the means of the apocarotenoids and the monoterpenes (Fig. 6a–d). The presence of a single major positive allele from ‘Covington’ suggests that this genotype may confer a positive effect on the accumulation of apocarotenoids and monoterpenes. This effect could potentially be governed by a single gene with pleiotropic effects or by different, closely linked genes within the colocalized QTL regions on LG 3 and LG 9.

In contrast, QTLs identified on LG 12 for the apocarotenoids and monoterpenes, showed a narrower support interval compared to those on LG 3 and 9 (Table 1), with the parents showing additive allelic effects. Except for the QTLs for β-ionone and (E)-rose oxide, the support interval for other apocarotenoids and monoterpenes on LG 12 were between 167.02–184.15 cM (marker S12_22117539–S12_23076980). NCDM04-001 contributed a major positive allele ‘i’ to the population means of the apocarotenoids on LG 12 (Fig. 6e–f). Covington also contributed some positive alleles to the apocarotenoids on this linkage group; however, the effect was slightly lower than that of NCDM04-001. The positive effects contributed by both parents suggest the potential for complementary or additive genetic effects at this region, although further validation would be needed to confirm the underlying genetic interactions. The combination of favorable alleles from both parents may therefore have potential value for breeding for these traits.

### Putative candidate genes underlying major QTL regions

Putative candidate gene analysis identified several genes located within or near QTL regions associated with apocarotenoid and monoterpene VOCs (Table S5). On LG 3, a NAC domain-containing gene overlapped with the β-ionone QTL peak at S3_764435, while *GGPS* (itf03g04380) and *PSY* (itf03g05110) were located within the QTL region. On LG 12, the α-cyclocitral QTL peaked at S12_22117539, which was approximately 4.5 kb from *IbOr* (itf12g24270). Several *CCD* and *NCED* genes were also identified within or near apocarotenoid QTL regions (Table S5). For monoterpene VOCs, seven terpene synthase genes were identified within the shared support region of the geraniol and linalool QTLs on LG 9. These genes were located between 2,839,581 and 2,918,485 bp and were annotated as linalool synthase or (3S)-linalool/(E)-nerolidol/(E,E)-geranyllinalool synthases. Candidate genes were prioritized based on their proximity to QTL peaks and annotated functions.

## Discussion

### Phenotypic variation and distribution of VOCs

A total of 176 VOCs differentiated the genotypes in the ‘DC’ mapping population, with sesquiterpenes identified as the most abundant group. This finding is consistent with previous studies that characterized VOCs in the same mapping population (Abugu et al. 2026) and in sweetpotato of varying flesh colors using GC × GC-ToFMS (Shen et al. 2024). In contrast, studies using one-dimensional GC–MS reported a higher abundance of compounds derived from Strecker degradation (SD) of amino acids in orange-, yellow-, and cream-fleshed sweetpotato roots (Yao et al. 2023; Zhang et al. 2023a, 2023b, 2021a). These differences may reflect variation in analytical platforms and VOC sampling and detection methods, which can influence the detection and relative abundance of VOCs.

For approximately 13% of the VOCs, some progeny exhibited phenotypic values beyond those observed in both parental lines, which may indicate transgressive segregation for those traits in the DC population. Distribution shapes also ranged from near-symmetrical to skewed, bimodal, or irregular patterns, reflecting non-uniform segregation of volatile abundance across the population. Similar segregation patterns have been previously reported in other crops. For example, Yang et al. 2023, reported transgressive segregation and a wide range of variation for 19 VOCs in progeny from an apple fruit mapping population. Similarly, Christeller et al. 2019 reported that, among 77 VOCs identified in apple peels, 9, 41 and 27 of them exhibited bimodal, skewed and normal distributions, respectively. Other studies using mapping populations in grape hybrids (Koyama et al. 2022) and tomato (Tieman et al. 2006) have also reported distribution patterns that depart from normality for flavor-associated VOCs. The variation in distribution patterns across compounds highlights the complex genetic architecture underlying VOC accumulation and indicates substantial phenotypic variation within the DC population for QTL analysis. Overall, these findings provide a phenotypic basis for investigating genomic regions associated with volatile composition and their potential application in breeding for flavor-related traits.

### Genetic architecture of VOCs and QTL distribution

The work presented here represents the first genetic study of VOC variation in sweetpotato. Overall, 85 QTLs were identified across the differentiating VOCs, including several regions associated with multiple compounds, suggesting both compound-specific and shared genetic effects on VOC accumulation in the DC population. Unlike previous QTL studies that have focused primarily on individual flavor-associated compounds, this study evaluated differentiating VOCs more broadly according to their potential biochemical precursors. This approach identified genomic regions associated with VOCs previously linked to flavor attributes as well as with other volatile compounds, providing a broader perspective on the genetic basis of VOC variation in sweetpotato. To further examine these associations, we next consider QTLs for VOCs derived from the monoterpene, sesquiterpene, and apocarotenoid pathways.

Correlation-based clustering grouped 39 sesquiterpene VOCs, for which 30 QTLs were identified, including 17 and 6 co-localizing on LGs 5 and 6, respectively. Similarly, several monoterpenes showed strong phenotypic correlations, with 5 QTLs co-localizing on LG 9. These patterns suggest that a limited number of genomic regions may contribute to the accumulation of multiple terpene VOCs. Similar clustering of terpene VOCs and their corresponding QTLs have been reported in citrus, apple, tomato, almond, and peach (Yu et al. 2017; Dunemann et al. 2009; Zanor et al. 2009; Sánchez-Perez et al. 2007; Eduardo et al. 2013). The co-localization of multiple terpene QTLs may reflect shared genetic regulation or closely linked genes, potentially including terpene synthase gene clusters that have arisen through tandem duplication (Jiang et al. 2019). However, phenotypic correlation did not consistently correspond to QTL co-localization. For example, (E)-rose oxide and nerol oxide were strongly correlated (r = 0.80), yet only (E)-rose oxide had a detected QTL, whereas weakly correlated geraniol and linalool (r = 0.39) mapped to overlapping regions. Such differences may reflect pathway branching, enzyme specificity, minor-effect loci, or environmental effects. Although geraniol and linalool share a common precursor, their formation is mediated by distinct enzymes and may therefore be differentially regulated (Iijima et al. 2004).

Several negative correlations were also observed among terpene VOCs, including D-limonene and α-terpinene (r = − 0.48), α-terpinene and β-cubebene (r = − 0.33), α-terpinene and ( −)-cyperene (r = − 0.32), D-limonene and rotundene (r = − 0.31), and α-terpinene and β-elemene (r = − 0.29). These inverse associations may reflect differences in precursor allocation or metabolic flux through branched terpene pathways, where multiple compounds can arise from shared precursor pools and interconnected biosynthetic routes (Lanier et al. 2023; Bergman and Dudareva 2024). Metabolic channeling and regulation at pathway branch points may further influence the relative accumulation of individual terpenes, although the mechanisms controlling flux between competing terpenoid pathways remain incompletely understood (Demurtas et al. 2022; Bergman et al. 2024). However, the semi-quantitative nature of the VOC measurements may also contribute to these associations. Thus, the negative correlations should be interpreted as statistical relationships rather than direct evidence of metabolic competition or trade-offs.

### Genetic architecture of flavor-associated VOCs

Of the 27 flavor-associated VOCs evaluated in this study, relatively few had detectable QTLs. For example, no QTLs were detected for major predictors of ‘caramel’ flavor, such as 2-furanmethanol. These VOCs, along with those derived from Strecker degradation and fatty acid oxidation, arise through complex biochemical and non-enzymatic reactions involving shared and competing precursors, which may contribute to their weak correlations and limited QTL co-localization (Kays & Wang 2000; Mastrocola et al. 2000; Troise et al. 2020). Unlike compounds that are more directly associated with specific biosynthetic pathways, the accumulation of these thermally derived VOCs depends on the availability and relative abundance of multiple substrates, as well as the reaction conditions during heating. Consequently, genetic variation affecting precursor pools may influence their abundance indirectly, while variation at multiple metabolic steps may have relatively small individual effects that are difficult to detect as QTLs. In addition, the same precursor may contribute to the formation of multiple products, resulting in metabolic competition and differences in flux among reaction pathways (Kays and Wang 2000). These factors may help explain why several flavor-associated VOCs showed limited QTL co-localization despite their importance as predictors of sensory attributes. Thus, the absence of detectable QTLs for some flavor-associated VOCs does not necessarily indicate a lack of genetic influence, but may instead reflect the complex and highly integrated nature of their formation.

In contrast, two QTLs were identified for each of the apocarotenoids β-ionone and α-cyclocitral and the monoterpenes geraniol and linalool, all of which have been associated with ‘cooked carrot’ flavor in sweetpotato. QTLs for β-ionone and α-cyclocitral have also been reported in tomato (Mathieu et al. 2009; Simkin et al. 2004), while QTLs for linalool and geraniol have been identified in carrot, grape, and tea tree (Keilwagen et al. 2017; Koyama et al. 2022; Voelker et al. 2023). These cross-species parallels suggest that aspects of volatile biosynthesis may be genetically conserved and support the biological relevance of the QTLs identified in sweetpotato. Together, these findings indicate that some flavor-associated VOCs may have more tractable genetic control than others, providing potential targets for genetic improvement of sweetpotato flavor.

The apocarotenoid QTLs identified in this study, span the same genetic and genomic regions with the QTLs previously reported for β-carotene (the main precursors of apocarotenoids) in the DC (Kinczyk 2019) and a Beauregard x Tanzania (BT) mapping population (Gemenet et al. 2020). Both studies identified two major β-carotene QTLs on LGs 3 and 12. Kinczyk (2019) reported ranges of 30.02–33.02 cM on LG 3 and 168.00–170 cM on LG 12; whereas Gemenet et al. (2020) reported broader ranges 0.00–121 cM on LG 3 and 124.12–176.02 cM on LG 12 (Table S4). The β-ionone QTL identified in our study (3.00–33.02 cM on LG 3 and 60.06–168 on LG 12) colocalized with the previously reported β-carotene QTLs, supporting the validity of our findings. Importantly, the monoterpene QTLs reported here represent the first of such findings in sweetpotato, underscoring the novelty and potential impact of this study for future flavor-focused breeding efforts.

‘Covington’ alleles exerted the largest positive effects for most apocarotenoid and monoterpene QTLs on LGs 3 and 9 (Fig. 6). In contrast, on LG 12, NCDM04-001 contributed allele ‘i’, which was associated with a positive effect on apocarotenoid abundance, while ‘Covington’ contributed a smaller positive effect. As previously reported for BC (Kinczyk 2019), the allelic effects observed for VOCs were consistent with additive effects. Thus, individuals carrying allele ‘d’ from ‘Covington’ on LG 3 and allele ‘i’ from NCDM04-001 on LG 12 may have the potential to accumulate higher levels of apocarotenoids. Similarly, individuals carrying allele ‘b’ from ‘Covington’ on LG 9 may have higher levels of monoterpenes associated with cooked carrot flavor. Prior consumer research in African populations suggests that a strong ‘cooked carrot’ flavor may not be desirable in sweetpotato (Laurie et al. 2013), although this has not been evaluated among US consumers. If this flavor is also found to be undesirable among US consumers, the favorable alleles associated with increased cooked-carrot-related VOCs could potentially be considered as selection targets for reducing this flavor attribute. However, these allelic effects should be considered preliminary because the QTLs were evaluated in a single location and season and have not been validated across environments or seasons. Further validation of these QTLs and their allelic effects, together with consumer acceptance studies in the US, will be necessary to determine whether these loci consistently influence cooked carrot flavor and can be reliably used in breeding.

### Biological relevance of putative candidate genes underlying flavor- associated QTLs

The identification of putative candidate genes within or near the QTL regions provided potential biological support for several loci associated with VOC variation. For the apocarotenoids, a NAC domain-containing gene overlapped the β-ionone QTL peak on LG 3. NAC transcription factors have been implicated in the regulation of carotenoid accumulation in both sweetpotato and other plant species. In sweetpotato, overexpression of *IbNAC29* increased β-carotene and other carotenoids and was associated with changes in the expression of carotenoid biosynthesis genes (Xing et al. 2023), while *SlNAC4* has been shown to positively regulate carotenoid accumulation in tomato (Zhu et al. 2014). These findings support the NAC gene identified in the present QTL region as a biologically plausible candidate for influencing carotenoid-derived VOC variation, although its identity and causal role remain to be confirmed. The co-occurrence of *GGPS* and *PSY* within the LG 3 QTL region further supports a potential connection between carotenoid biosynthesis and β-ionone accumulation. Similarly, the proximity of *IbOr* to the α-cyclocitral QTL on LG 12 is notable because this gene has previously been associated with β-carotene accumulation in sweetpotato (Gemenet et al. 2020; Kinczyk 2019). Because α-cyclocitral is derived from carotenoid degradation, the co-localization of carotenoid-associated genes and apocarotenoid QTLs supports a potential genetic relationship between carotenoid metabolism and the production of carotenoid-derived VOCs. However, these positional associations do not establish that the identified genes are responsible for the observed QTL effects.

For the monoterpene VOCs, seven terpene synthase genes were identified within the shared support region of the geraniol and linalool QTLs on LG 9. Terpene synthases catalyze the formation of diverse terpenoid compounds, and genes involved in linalool and geraniol biosynthesis have been characterized in other plant species (Lavy et al. 2002; Van Schie et al. 2007; Zhou et al. 2020). The presence of multiple terpene synthase genes within this QTL region therefore provides a plausible biological basis for the observed variation in geraniol and linalool. Together, these candidate genes provide biologically plausible targets within VOC-associated QTL regions and a foundation for future studies integrating gene expression, fine mapping, and functional validation to determine their contributions to VOC accumulation.

### Limitations of current study and pathways for further research

The limitations of this study include the evaluation of the progeny at a single location during one growing season. Although previous studies have reported relatively low genotype × environment (G × E) interactions for several sweetpotato quality traits, including dry matter, starch, β-carotene, and sugars, across environments (Cervantes-Flores et al. 2011; Yada et al. 2017), the environmental stability of VOC-associated QTLs remains unknown. This is particularly relevant for VOC traits because volatile accumulation can be influenced by environmental conditions during plant development and by postharvest physiological processes that affect precursor availability and metabolic flux. Consequently, QTLs identified in the present study may differ in their detectability, estimated effect sizes, or expression across environments. Environmental variation could reduce the power to detect QTLs with relatively small effects in some environments, while also producing environment-specific QTLs that may not be consistently detected across locations or growing seasons. Thus, the QTLs reported here should be interpreted as loci associated with VOC variation under the specific environmental conditions evaluated rather than as universally stable determinants of VOC accumulation. Variation in starch and sugar content may also influence VOC accumulation through differences in precursor availability; however, these traits were not measured in the present study and therefore their contribution to environmental variation in VOC profiles could not be evaluated.

Future studies should therefore evaluate the stability of the identified VOC-associated QTLs across multiple environments and growing seasons, ideally including at least two growing seasons and multiple locations representing contrasting production environments. Multi-environment trials using a subset of genotypes from the DC population would provide a practical first step for assessing the reproducibility and stability of the most promising QTLs, while validation in an independent genetic population would provide stronger evidence for their broader utility. Particular priority could be given to QTLs with high LOP scores and those that overlap across related compound classes, as these loci may represent particularly robust genomic regions influencing VOC accumulation. Comparing QTL detection, effect sizes, and allele × environment interactions across environments would help distinguish stable QTLs from environment-specific loci and provide a more accurate assessment of their potential utility for sweetpotato flavor improvement. In parallel, measurement of starch, sugars, and other relevant metabolic precursors across environments could help clarify the mechanisms underlying environmental effects on VOC accumulation. Further validation of the underlying candidate genes through fine mapping, transcriptomic analyses, and functional studies will also be necessary to establish the biological mechanisms connecting these loci to VOC production and flavor-related phenotypes.

## Conclusion

This study provides new insight into the genetic architecture of VOC variation in sweetpotato. Volatile profiles were characterized using GC × GC-ToFMS in an NCDM04-001 × ‘Covington’ mapping population, and the genetic basis of VOC variation was investigated. A total of 85 QTLs were identified for 61 volatile compounds across 10 linkage groups. Several QTLs co-localized for compounds sharing similar biochemical precursors, with multiple genomic hotspots suggesting the potential involvement of linked genes and/or shared biosynthetic pathways. These patterns indicate that VOCs may be governed by both shared and compound-specific genetic factors. In particular, QTLs identified for monoterpenes and apocarotenoids associated with ‘cooked carrot’ flavor provide promising targets for further investigation of the genetic basis of this flavor attribute. The allelic effects observed for some QTLs were consistent with additive effects, although the genetic architecture varied among compounds.

To our knowledge, this represents the first genetic study of VOC variation in cooked sweetpotato and the first report of QTLs associated with these compounds. The QTLs and functionally annotated genes identified in this study provide a foundation for investigating the genetic control of flavor-associated VOCs and developing markers for future breeding applications. However, validation across multiple environments, seasons, and populations, together with consumer acceptance studies, will be necessary to establish the stability and breeding utility of these loci. Overall, this study provides a framework for further investigating the genetic and environmental factors underlying VOC variation and flavor quality in sweetpotato.

## Supplementary Information

Below is the link to the electronic supplementary material.

Supplementary file1 (DOCX 6232 KB)

Supplementary file2 (XLSX 1291 KB)

## Data Availability

The datasets generated and analyzed during the current study are available in the Zenodo repository at https://doi.org/10.21203/rs.3.rs-9905073/v1.
