# Supplementary material for: A single-environment QTL analysis of flavor-related volatile compounds in a sweetpotato mapping population
Source: Euphytica. 2026 Nov 3;222(12):181. doi: 10.1007/s10681-026-03810-w (PMC13731720; doi:10.1007/s10681-026-03810-w)
Supplement: Supplementary file 1 [file 10681_2026_3810_MOESM1_ESM.docx]

## Supplemental Figures

**Fig S1**: Pie chart indicating the number of VOCs identified in the DC population, grouped according to their mechanism of formation. See Table S3 for a detailed list of compounds.

VOCs (log _10_ relative peak area)

**Fig S2 continued:** Frequency distribution of the 176 identified VOCs in the DC population. Each gray bar represents the frequency of progeny for a given VOC, with the x-axis indicating the abundance of VOCs (log10 relative peak area) and the y-axis showing the number of progenies Red and blue dashed lines indicate the parental values for ‘Covington’ and NCDM04-001, respectively

**Fig S2 continued:** Frequency distribution of the 176 identified VOCs in the DC population. Each gray bar represents the frequency of progeny for a given VOC, with the x-axis indicating the abundance of VOCs (log10 relative peak area) and the y-axis showing the number of progenies. Red and blue dashed lines indicate the parental values for ‘Covington’ and NCDM04-001, respectively

**Fig S2 continued:** Frequency distribution of the 176 identified VOCs in the DC population. Each gray bar represents the frequency of progeny for a given VOC, with the x-axis indicating the abundance of VOCs (log10 relative peak area) and the y-axis showing the number of progenies Red and blue dashed lines indicate the parental values for Covington’ and NCDM04-001,respectively

**Fig S2 continued:** Frequency distribution of the 176 identified VOCs in the DC population. Each gray bar represents the frequency of progeny for a given VOC, with the x-axis indicating the abundance of VOCs (log10 relative peak area) and the y-axis showing the number of progenies Red and blue dashed lines indicate the parental values for Covington’ and NCDM04-001, respectively

**Fig S2 continued:** Frequency distribution of the 176 identified VOCs in the DC population. Each gray bar represents the frequency of progeny for a given VOC, with the x-axis indicating the abundance of VOCs (log10 relative peak area) and the y-axis showing the number of progenies Red and blue dashed lines indicate the parental values for Covington’ and NCDM04-001, respectively

**Fig S2 continued:** Frequency distribution of the 176 identified VOCs in the DC population. Each gray bar represents the frequency of progeny for a given VOC, with the x-axis indicating the abundance of VOCs (log10 relative peak area) and the y-axis showing the number of progenies Red and blue dashed lines indicate the parental values for Covington’ and NCDM04-001, respectively

VOCs (log _10_ relative peak area)

VOCs (log _10_ relative peak area)

VOCs (log _10_ relative peak area)

**Fig S3:** Plot representing all QTLs identified for the VOCs in the DC population. The heat map represents the significance level and the size of the circle represent the PVE (phenotypic variation explained).

**Fig S4:** QTL plot for VOCs whose mechanism of formation is uncharacterized. -Log10(P) represent the logarithm of P-values for every genomic position using the final QTL model for each trait.

**Fig S5:** QTLs plot for the VOCs with unknown identity. -Log10(P) represent the logarithm of P-values for every genomic position using the final QTL model for each trait.
